# Reporting of interventions in randomised trials: an audit of journal Instructions to Authors

**DOI:** 10.1186/1745-6215-15-20

**Published:** 2014-01-14

**Authors:** Tammy Hoffmann, Thomas English, Paul Glasziou

**Affiliations:** 1 Centre for Research in Evidence-Based Practice, Faculty of Health Sciences Medicine, Bond University, 4229 Queensland, Australia; 2 School of Health and Rehabilitation Sciences, University of Queensland, Brisbane, Australia

**Keywords:** Intervention reporting, Randomised controlled trial reporting, CONSORT

## Abstract

**Background:**

A complete description of the intervention in a published trial report is necessary for readers to be able to use the intervention, yet the completeness of intervention descriptions in trials is very poor. Low awareness of the issue by authors, reviewers, and editors is part of the cause and providing specific instructions about intervention reporting to authors and encouraging full sharing of intervention materials is important. We assessed the extent to which: 1) journals’ Instructions to Authors provide instructions about how interventions that have been evaluated in a randomised controlled trial (RCT) should be reported in the paper; and 2) journals offer the option of authors providing online supplementary materials.

**Methods:**

We examined the web-based Instructions to Authors of 106 journals (the six leading general medical journals, 50 randomly selected journals from the National Library of Medicine’s Core Clinical Journals, and 50 randomly selected journals from the remainder of the journal collection indexed by PubMed). To be eligible, each journal must have published at least one randomised trial involving human participants each year from 2008 to 2012. We extracted all information related to the reporting of interventions, reporting of randomised trials in general, and online supplementary materials.

**Results:**

Of the 106 journals’ Instructions to Authors, only 15 (14%) specifically mentioned the reporting of interventions and most of these provided non-specific advice such as ‘describe essential features’. Just over half (62, 58%) of the journals mentioned the Consolidated Standards of Reporting Trials (CONSORT) statement in their author instructions. Seventy-eight (74%) of the journals’ instructions mentioned the option of providing supplementary content online as part of the paper; however, only four of these journals explicitly encouraged or mandated use of this option for providing intervention information or materials.

**Conclusions:**

Most journals’ Instructions to Authors do not provide any specific instructions regarding reporting of interventions or encourage authors to provide online supplementary materials to enhance intervention reporting. Journals can help to improve the problem of incomplete intervention reporting by providing specific instructions to authors and peer reviewers about intervention reporting and requiring full intervention descriptions to be provided.

## Background

A complete description of the intervention in a published report is necessary for the reader of the study to be able to use the intervention. Even for studies which conclude that an intervention is not effective, complete intervention descriptions are needed for other researchers to replicate and build on these findings.

A number of studies have highlighted the problem of incomplete reporting of interventions; all concluding that intervention descriptions are woefully inadequate [1-5]. For example, a recent analysis of the completeness of descriptions of 137 non-pharmacological interventions in randomised trials published in the six leading general medical journals found that only 39% of the interventions were adequately described in the primary paper and associated materials [1]. Analysis of a sample of trials and systematic reviews of high quality and clinical relevance found that only 49% contained complete intervention descriptions, with complete intervention reporting worse for non-pharmacological interventions than pharmacological interventions [2].

The publication of incomplete intervention descriptions is likely a result of several factors, including an inadequate peer review process and low awareness of the issue by authors, reviewers, and editors [6]. Another potential barrier is that the complete description of an intervention involves providing a full description of, or access, to all intervention materials. Intervention materials are crucial to the replicability of interventions but are frequently missing from intervention descriptions [1]. Providing intervention materials via the supplementary material offered by many journals is one possible solution, yet the frequency that this is encouraged by journals has not been examined.

Providing authors with specific guidance for research reporting can improve the quality of reporting. Completeness of the reporting of randomised trials has improved since the introduction and journal endorsement of the Consolidated Standards of Reporting Trials (CONSORT) statement [7,8]. Despite advancements in research reporting standards and publication of numerous reporting standards, the quality of intervention reporting has received comparatively little attention. Exploration of the instructions that journals provide to authors regarding how to describe interventions in publications has not occurred. We aimed to assess the extent to which: 1) journals’ Instructions to Authors provided instructions about how interventions that have been evaluated in a randomised trial should be reported in the paper; and 2) journals offer the option of authors providing online supplementary materials.

## Methods

### Sample of journals’ Instructions to Authors

The sample consisted of 106 journals, in three groups. One group contained the six leading general medical journals identified by citation impact factors from the ISI Web of Knowledge in 2011 (*New England Journal of Medicine*, *JAMA*, *The Lancet*, *Annals of Internal Medicine*, *PLOS Medicine*, *BMJ)*. The second group contained 50 randomly selected journals from the National Library of Medicine’s Core Clinical Journals (http://www.nlm.nih.gov/bsd/aim.html). The list of journal identification numbers was exported into Excel (Microsoft, Redmond, WA, USA), a computer-based random number generator was used to select journals, and the eligibility of each journal was checked by one author (TH). To be eligible for inclusion, each journal must have published at least one randomised trial involving human participants per year for a 5-year period commencing in 2008. Journals which did not provide instructions to authors in English were also excluded. Journals that did not meet these criteria were excluded and replaced by the next in the random sequence. This occurred 24 times for the core collection journals.

The third group contained 50 randomly selected journals from the remainder of the journal collection indexed by PubMed (referred to throughout as non-core collection). In PubMed each year, from 2008 to 2012, was searched separately using the following example search strategy: (2011 [dp] AND English[la]) NOT jsubsetAIM). Endnote and pivot tables in Excel were used to manipulate the data into a suitable format and the remainder of the procedure was the same as described for the core collection journals. Journals were excluded and replaced by the next in the random sequence 72 times for the non-core collection.

### Audit of journals’ published Instructions to Authors

For each eligible journal, two authors (TH and TE) independently located and read, between November 2012 and February 2013, the journal’s Instructions to Authors on the journal’s website. To ensure that relevant information was not missed, instructions were also searched using relevant text words (for example, treatment, intervention, procedure, online, supplementary, multimedia, video, additional, material, RCT, randomized, randomised, CONSORT, trial, extension statement, guideline, checklist, appendix, appendices). The same two authors independently extracted all information using a data extraction form developed in Excel related to: the reporting of interventions (specific to randomised trials and also general advice for reporting research articles); reporting of randomised trials in general; reporting of interventions in the abstract; and the journal’s instructions regarding online supplementary materials. Relevant text was extracted from each website and then coded (for example according to the categories in Table 1). Any disagreements about data extraction were resolved through discussion between the two assessors.

**Table 1:** Journals’ Instructions to Authors regarding reporting of randomised trials and/or interventions

**Content of journals’ Instructions to Authors of randomised trials**	**Leading general medical journals (n = 6)**	**Core collection journals (n = 50)**	**Non-core collection journals (n = 50)**	**Total (n = 106)**
Use CONSORT statement	6 (100)	21 (42)	35 (70)	62 (58)
Use the appropriate CONSORT extension statement	5 (83)	1 (2)	0 (0)	6 (6)
Specific mention about reporting of interventions	3 (50)	5 (10)	7 (14)	15 (14)
A statement that ‘methods should be described in a way that enables reproduction of findings’	2 (33)	10 (20)	7 (14)	19 (18)
No specific instructions about the reporting of interventions or randomised trials	0	19 (38)	12 (24)	31 (29)

Results are presented as numbers (percentages). CONSORT, Consolidated Standards of Reporting Trials.

## Results

### Instructions regarding reporting interventions in randomised trials

Of the 106 journals’ Instructions to Authors, only 15 (14%) specifically mentioned the reporting of interventions (Table 1). However, the majority (62, 58%) of journals mentioned the CONSORT statement in their author instructions, with a few journals (6, 6%) also mentioning that a CONSORT extension statement should be used where appropriate. A small number of journals’ instructions (19, 18%) provided a general instruction that the study methods should be described in a way that enables the findings to be reproduced, but did not specifically mention interventions as part of this advice. Just under one-third (29%) of journals’ Instructions to Authors did not provide any advice about how randomised trials, and/or interventions, should be reported.

Of the 15 journals that specifically mentioned the reporting of interventions, the instructions ranged from non-specific statements about describing ‘essential features of interventions’ or ‘clear descriptions’ to more detailed guidance. Table 2 contains the verbatim instructions provided by each of these journals.

**Table 2:** Verbatim examples of intervention reporting advice from journals’ Instructions to Authors

**Type of instruction**	**Verbatim examples**
**Non-specific instructions**	“Essential features of interventions …’ *(in three journals)*”
	“Describe study procedures, including any interventions …”
	“A clear description of all interventions and comparisons …”
	“… should give a description of the treatment, intervention, technique or procedure…”
	“… include enough information about the intervention(s) and comparator(s) (even if this was usual care) for reviewers and readers to understand fully what happened in the study.”
	“Describe the intervention itself and implementation …”
**Instructions that recommend providing the intervention manual**	“It is essential that reports of trials provide sufficient details on interventions so that readers can judge the applicability and clinical relevance of results. Authors are encouraged to provide a trial treatment manual as an online-only appendix.”
	“Evaluations involving behavioural interventions must include full manuals or protocols (or at least very detailed descriptions) of those interventions as supplementary files to be included published with the online version of the article.”
	“We will only consider protocols relating to interventions in which there is a commitment to public sharing of the intervention content in full.”
**Instructions that suggest specific elements of the intervention should be reported**	“The essential features of any interventions should be described, including their method and duration of administration. The intervention should be named by its most common clinical name, and non-proprietary drug names should be used.”
	“Any instruments or drugs (including contrast) utilised should be identified with trade names and manufacturer’s name and location in parentheses. Procedures should be described in sufficient detail to allow others to reproduce the study.”
	“Intervention reports are … allowed 4 additional pages over the 16 page limit for detailed description. Provide extensive details regarding any interventions (see Conn, WJNR 34, 427–433 for intervention details to report).”
	“Any treatment (including surgery) should be briefly described, particularly when surgeons apply unique approaches or when all patients did not undergo essentially identical procedures. Previously described approaches require only brief mention with citations to those methods. All relevant aspects of post treatment follow-up care should be described…. Authors must note whether the treatment was uniform among all patients or varied. If varied, they should specify the indications for treating patients in varying ways…”
	“Identify the methods, apparatus (manufacturer’s name in parentheses), and procedures in sufficient detail to allow other researchers to reproduce the results. Identify precisely all drugs and chemicals used, including generic names, dosages, and routes of administration. If trade names for drugs and chemicals are included, give the manufacturer’s name and location.”

A small number (16, 15%) of journals also specifically mentioned that the intervention should be described in the abstract. This occurred in five (83%) of the leading general medical journals, seven (7%) of the core collection journals, and four (4%) journals from the non-core collection. For most journals, this mention was limited to a single word (‘intervention’) when describing what should be included in the Methods section of an abstract for a randomised trial. For five journals, some elaboration was provided. For example, ‘interventions: what, how, when and for how long’ , ‘the essential features of any interventions should be described, including their method and duration of administration’ , and ‘describe the … intervention … used’.

### Instructions regarding online supplementary material

Seventy-eight (74%) of the journals’ instructions mentioned the option of providing supplementary content online as part of the paper. This option was offered by all six (100%) of the leading general medical journals, 39 (78%) of the core collection journals, and 33 (66%) of the non-core collection journals. Only four of these journals explicitly encouraged or mandated use of this option for providing intervention information or materials, and one encouraged this for providing more detailed information about the methodology, not specifically the intervention. The below list provides verbatim examples of instructions for providing intervention information as supplementary material:

• “To enable readers to replicate your work or implement the interventions in their own practice please also provide (uploaded as one or more supplemental files, including video and audio files where appropriate) any relevant detailed descriptions and materials. Alternatively, please provide in the manuscript, URLs to openly accessible websites where these materials can be found.”

• “To help the reader … submit study protocols, treatment manuals, detailed descriptions of evaluation and intervention procedures, treatment progression algorithms, etc. These can be submitted as online-only tables, figures, appendixes, or video clips. They are reviewed by the editors and Editorial Board and should be submitted at the same time that the manuscript is submitted.”

• “Authors are encouraged to provide a trial treatment manual as an online-only appendix.”

• “Evaluations involving behavioural interventions must include full manuals or protocols (or at least very detailed descriptions) of those interventions as supplementary files to be included published with the online version of the article.”

• “Detailed methodology or supporting information relevant to the methodology can be published on our Web site.”

## Discussion

This audit of the Instructions to Authors from a sample of 106 journals found that very few journals provide instructions about how to report interventions in randomised trials. In addition to low awareness by authors, reviewers, and editors of the importance of complete intervention reporting [6], this lack of instruction may also be contributing to the problem of poor reporting of interventions. Providing guidance to authors as part of their Instructions to Authors may be a way of increasing the completeness of intervention descriptions that are submitted. As the majority of problems with incomplete intervention descriptions are not detected by peer reviewers and editors [6], building similar guidance into the peer review system is also important, and may improve reviewer and editor performance in this area.

Just over half (58%) of the journals instructed that the CONSORT statement should be followed when reporting randomised trials. CONSORT contains an item (item 5) about how interventions should be reported and provides general advice to report ‘the interventions for each group with sufficient details to allow replication, including how and when they were actually administered’ [7]. However the CONSORT statement does not provide specific guidance about all the elements of an intervention that are needed to enable replication of a trial’s intervention. This includes elements such as: the components of the intervention, who delivered the intervention, where the intervention was delivered, the schedule of the intervention, and any tailoring or standardisation of the intervention [9]. In general, the topsix journals had more detailed instructions, but none had specific instructions for interventions. Of the few journals that did specifically mention intervention reporting, most provided non-specific advice such as ‘provide a complete description’ or ‘describe essential features’. Such advice is not sufficient to guide authors in providing a complete description as many are unaware of what comprises a good description. A new reporting guideline, TIDieR (Template for Intervention Description and Replication), which is an extension to item 5 of the CONSORT statement and item 11 of the SPIRIT statment, has recently been developed to provide detailed guidance for reporting interventions and its publication is forthcoming. The process of its development involved a literature review for relevant checklists and research, a Delphi survey of an international panel of experts to guide item selection, and a face-to-face panel meeting.

There are some CONSORT extension statements that provide detailed advice for reporting intervention details for particular intervention categories (for example, non-pharmacological interventions [10], herbal medicine [11], e-health interventions [12], homeopathy [13]). However, very few (6%) journals’ Instructions to Authors advised authors to use CONSORT extension statements when appropriate. A survey of peer reviewer instructions also found little mention of CONSORT extension statements [14], as did a survey which studied endorsement of the CONSORT statement by high impact factor medical journals [15]. Only 18% of journals’ instructions contained any mention of describing the methods in a way that could enable reproduction of the study. This is similar to the finding from a 2003 cohort of medical journals’ Instructions to Authors in which only 11% contained a comment about providing enough information to permit replication [16].

Almost 15 years ago, the opportunities afforded by electronic publishing were discussed and journals were encouraged to use it to improve the quality of reporting [17]. In particular, the scope to provide fuller descriptions of studies and related materials (such as trial protocols) were highlighted. A longitudinal study that examined the use of supplementary materials by medical journals over a 12-year period found that the percentage of articles that contained supplementary material increased from 7% in 2003 to 25% in 2009 [18]. However, this study did not specifically investigate whether online supplementary materials were used to provide intervention details. Despite the majority (74%) of journals in our sample listing the option of providing online supplementary materials, very few (5% of these journals) specifically encouraged or mandated use of this option for providing intervention information or materials. Online supplementary materials are ideally suited to providing intervention information and materials. Multiple formats can be used (such as videos, photos, large appendices, interactive material) to provide information that cannot be otherwise captured adequately in the text of a paper. This can be particularly useful for demonstrating aspects of interventions that are hard to describe, such as surgical techniques or manipulative physiotherapy techniques. Providing additional detail and materials as supplementary online materials also overcomes the word length barrier that is set by many journals.

Journals should modify their Instructions to Authors and encourage authors to use the online supplementary materials option to provide full descriptions of interventions, including any materials that were used as part of the intervention materials (for example, intervention manuals, patient booklets, training materials). An analysis of the completeness of reporting of 137 non-pharmacological interventions found that sufficient information about, and access to, intervention materials was the most frequently missing element of intervention descriptions; missing for 53% of interventions [1]. However, by contacting the trial authors and asking for additional information/materials, this element of an intervention description could be improved the most; to missing in only 8% of interventions [1]. Authors frequently have the materials, and are willing and able to share them. Journals introducing a requirement for all intervention materials to be provided in conjunction with the primary paper would increase the availability of these materials and enable replication of interventions. Currently only a few journals have an editorial policy that requires this [19] and we encourage more to adopt similar policies. A journal requirement that interventions must be completely described may be all that is required for some authors to provide this; for others, additional guidance about how to completely describe an intervention may also be required.

This study relied only on journals’ publically available Instructions to Authors and did not survey journal editors about their policies or guidance about intervention reporting. It has been noted previously that there can be inconsistency between journals’ editorial policies and the information provided in their Instructions to Authors [15]. It is possible that some journals provide specific advice to peer reviewers about the reporting of interventions; any such advice was not captured by auditing journals’ Instructions to Authors.

## Conclusions

Most journals’ Instructions to Authors do not provide any specific instructions regarding reporting of interventions. Similarly, the opportunities afforded by using online supplementary materials to assist in providing complete intervention descriptions are not being embraced by most journals, with very few journals instructing or suggesting authors use this option to enhance intervention reporting. The problem of incomplete intervention reporting is widespread, has the serious consequence of the research being unusable, and requires attention by all stakeholder groups. One way that journals can help in improving this problem is to provide specific instructions to authors and peer reviewers about how to report interventions (including using appropriate CONSORT extension statements where they exist), require full intervention descriptions to be provided, and encourage authors to use online supplementary materials, where appropriate, to achieve this.

## Abbreviations

CONSORT: Consolidated standards of reporting trials; NHMRC: National health and medical research council; RCT: Randomised controlled trial; SPIRIT: Standard protocol items: Recommendations for interventional trials; TIDieR: Template for intervention description and replication.

## Competing interests

TH and PG are members of a group that have prepared a CONSORT extension statement and checklist (The Template for Intervention Description and Replication (TIDieR) checklist) that provides specific advice about the reporting of interventions.

## Authors’ contributions

TH and PG conceived of the study and its design. TH and TE collected and analysed the data. TH coordinated the study and wrote the draft of the paper, with assistance from PG and TE. All authors read and approved the final manuscript.
